# Microtensile Bond Strength and Failure Mode of Different Universal Adhesives on Human Dentin

**DOI:** 10.1016/j.identj.2024.04.009

**Published:** 2024-05-11

**Authors:** Flor Santander-Rengifo, Carmen Martin Carreras-Presas, Rosa Aroste-Andía, Emily Hernández-Huamaní, Percy Gavilán-Chávez, Luis Cervantes-Ganoza, César Cayo-Rojas

**Affiliations:** a Doctoral Program and Health Sciences, Doctoral and Research School, Universidad Europea de Madrid, Madrid, Spain; b Academic Program of Dentistry, Universidad Peruana de Ciencias Aplicadas, Lima, Peru; c Head of Esthetic Dentistry Program, Faculty of Health and Biomedical Sciences, Dentistry Department, Universidad Europea de Madrid, Madrid, Spain; d School of Stomatology, Universidad Privada San Juan Bautista, Ica, Peru; e Faculty of Stomatology, Universidad Inca Garcilaso de la Vega, Lima, Peru

**Keywords:** Universal adhesives, Microtensile bond strength, Self-etch adhesives, Dentin

## Abstract

**Aim:**

The aim of this study was to compare the microtensile bond strength (µTBS) and failure mode of 4 different universal adhesive systems (UAs) on human dentin.

**Materials and methods:**

The study sectioned the occlusal thirds of 32 human third molars and divided them into 4 groups based on the adhesive system used. Group A: Palfique Universal Bond, Group B: Single Bond Universal, Group C: All-Bond Universal, and Group D: One Coat 7 Universal**.** The specimens underwent a 10,000-cycle thermocycling ageing process prior to testing (n = 32). Afterwards, 8 beams were obtained per group and subjected to µTBS testing using a digital universal testing machine at a speed of 1 mm/min. The microtensile bond strength values were analysed in Megapascals (MPa), and the failure mode was evaluated using a stereomicroscope. Welch's parametric ANOVA with robust variance and the Games-Howell post hoc test were used for µTBS comparisons, and Fisher's exact test was used to determine the association between adhesive type and failure mode. The significance level was set at *P* < .05.

**Results:**

Group D showed a significantly higher µTBS than groups A (*P* < .001) and B (*P* < .001), but no significant difference was observed with group C (*P*= .075). Furthermore, groups B and C showed significantly higher µTBS than group A (*P*< .001 and *P* < .001, respectively), but there was no significant difference between groups B and C (*P* = .132). Additionally, group A exhibited a significant association with an adhesive failure mode (*P* < .05), whereas groups B, C, and D were significantly associated with a mixed failure mode (*P* < .05).

**Conclusion:**

The One Coat 7 Universal adhesive system showed higher microtensile bond strength values and higher chemical interaction with dentin compared to Palfique Universal Bond and Single Bond Universal. However, no significant differences were observed compared to All-Bond Universal.

## Introduction

The pursuit of enhancing dental materials has resulted in extensive research on dental adhesive systems in recent decades. The objective is to create products that ensure long-term bonding and simplify the technique.^1^, ^2^, ^3^

The development of new adhesive systems has enabled their use in total etch, self-etch, or selective etch modes. These adhesives are commonly referred to as “universal” because they can be applied in different bonding strategies.^2^^,^^4^^,^^5^ The success of universal adhesive systems in the all-in-one self-etch mode depends on their ability to partially dissolve the smear layer without excessively demineralising the tooth surface or removing hydroxyapatite at the interface. The preservation of hydroxyapatite allows for calcium to chemically bond with the functional monomer.^2^^,^^6^

Universal self-etch adhesive systems contain hydrophilic functional monomers with carboxylic and phosphate groups. These groups act as proton donors during the partial demineralisation of the smear layer and have the potential to ionically bind with calcium in the residual hydroxyapatite (HAp).^7^ Functional monomers have a polymerisable group and a functional group. The functional group is a polar chemical group that can serve various purposes, such as wetting, demineralisation, and ionic bonding to the tooth substrate.^1^^,^^3^^,^^8^ The interaction potential between the functional monomer of an adhesive system and hydroxyapatite varies among different functional monomers. This difference explains the superior performance of self-etch adhesives, which leads to a longer-lasting final restoration.^3^^,^^7^

The functional monomer 10-methacryloyloxide dihydrogen phosphate (MDP) forms stable calcium phosphate complexes and regular layers on the surface of hydroxyapatite.^8^ The presence of long, hydrophobic 10-MDP chains confers hydrolytic stability and low solubility of the 10-MDP-Ca calcium salt that forms on the surface of hydroxyapatite, achieving an effective and durable bond with dentin.^1^, ^2^, ^3^^,^^9^ Micromechanical interlocking by optimising dentin hybridisation through resin tags and hybrid layer has been proposed to enhance the bond strength of self-etch adhesive systems.^2^^,^^10^

Another functional monomer is the 3-dimensional self-reinforcing acid (3D-SR). This monomer contains several functional groups per molecule, which allows for multiple point interactions with calcium in dentin.^1^^,^^7^ As a result, it creates strong adhesion to the tooth structure and 3-dimensional crosslinking through its reaction with calcium. The adhesive monomers’ crosslinks play a crucial role in the 3-dimensional crosslinking density through copolymerisation reactions, resulting in a uniform, thin, and highly adhesive layer. The manufacturer also claims that stabilising the adhesive system's pH at 2.8 reduces the hydrolysis of the monomers during storage.^1^^,^^11^

The interaction between adhesive systems and tooth structure can be significantly influenced by their components. However, there is limited research on the actual interaction of existing functional monomers with dentin and their effects on bonding.^1^ Thus, this study aimed to compare the microtensil bond strength and failure mode of 4 different universal adhesive systems on human dentin. The null hypothesis was that there were no significant differences in microtensil bond strength and failure mode between the 4 universal adhesive systems and human dentin.

## Materials and methods

### Ethical considerations

This study received approval from the ethics and research committee of the Universidad Europea de Madrid with internal code CIPI/22.331. The teeth used in this study were voluntarily donated by patients treated at the oral and maxillofacial surgery service of the Universidad Peruana Cayetano Heredia for research purposes under informed consent and with respect for the confidentiality of the participants and the Declaration of Helsinki.^12^

### Study design

The study was conducted experimentally in vitro at the Oral Health Research Laboratory (LISO) of the Faculty of Stomatology at the Universidad Peruana Cayetano Heredia and at the High Technology Certified Laboratory (ISO/IEC Standard: 17025), Peru, from December 2022 to February 2023. The CRIS Guidelines (Checklist for Reporting In-vitro Studies) were followed.^13^

### Sample calculation and selection

**Fig. 1:**
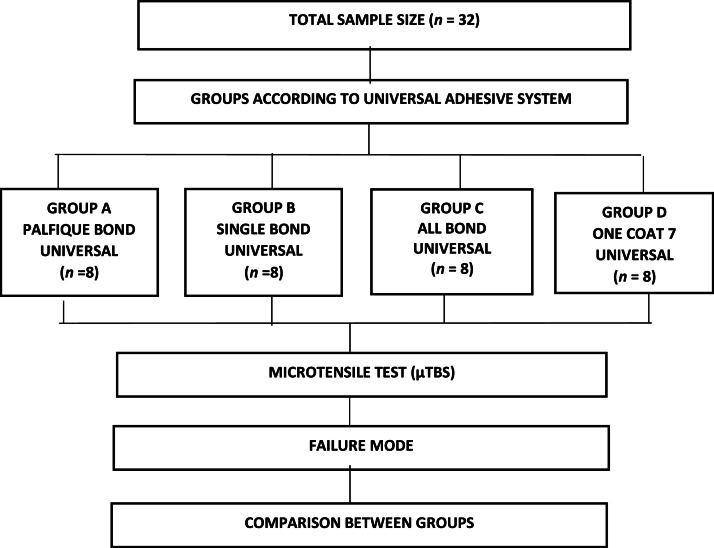
Random distribution of groups according to sample size.

A total of 32 sample units were distributed into 4 different groups of universal adhesive systems using a beam design (n = 8). The minimum sample size was calculated using G*Power version 3.1.9.7 statistical software. The significance level (α) was set at 0.05, the statistical power (1 - β) at 0.80, and the effect size at 2.3. All parameters were obtained from previous studies.^5^^,^^6^^,^^14^ The experimental groups were classified based on the universal adhesive systems used (Table 1 and Figure 1):

• Group A: Palfique Universal Bond (Tokuyama Dental Corporation)
• Group B: Single Bond Universal (3M ESPE)
• Group C: All-Bond Universal (Bisco)
• Group D: One Coat 7 Universal (Coltene)

**Table 1:** Technical profile of products used.

Adhesive, batch, manufacturer	pH	Composition	Application mode (Self-etching)
Palfique Universal Bond. (Tokuyama Dental Corporation, Tokyo, Japan; 203E82)	Mild 2.0	Bottle 1 (Bond):3D-SR, MTU-6, HEMA, BIS-GMA, TEGDMA, Water. Bottle 2:	1. Adhesive from 2 bottles (bottle A and B). Dispense 1 drop per bottle, mix and rub for 5 sec.
		Y-MPTES, Borate, Peroxide, Acetone, Isopropyl alcohol and Water.	2. Air dry the adhesive for 5 sec. to allow the solvent to evaporate.
Single Bond Universal (3M ESPE, St. Paul, MN, USA; 20503A)	Mild 2.7	10-MDP, HEMA, silane, dimethacrylate resin, VitrebondTM copolymer, fibres, ethanol, water and initiators.	1. Apply the adhesive and rub for 20 sec.
			2. Air dry the adhesive for 5 sec. to allow the solvent to evaporate.
			3. Light cure for 10 sec. at 1200 mW/cm^2^.
All-Bond Universal, (Bisco, Schaumburg, IL, USA, 2200004058)	Ultra-mild 3.1	Bis-GMA, 10-MDP, HEMA, ethanol, Initiators and water.	1. Apply 2 separate coats of adhesive with agitation for 10-15 s per coat.
			2. Air dry the adhesive for 10 s to allow the solvent to evaporate. No movement of the adhesive should be visible.
			3. The surface should be uniform and glossy in appearance. If not, repeat steps 1 and 2.
			4. Light cure for 10 s. at 1200 mW/cm^2^.
One coat 7 Universal (Coltene, Altstätten, Suiza; J60935)	Mild 2.8	Methacrylates, HEMA, 10-MDP, photoinitiators, ethanol, water.	1. Apply the adhesive and rub for 20 sec.
			2. Gently air dry the adhesive for 5 sec. to allow the solvent to evaporate.
			3. Light cure for 10 sec. at 1200 mW/cm^2^.
Material	Composition		Instructions for use
Filtek Z350 XT nanohybrid (3M ESPE, St. Paul, MN, USA; NF15889)	Matrix: BIS-GMA, UDMA, BIS-EMA, PEGDMA and TEGDMA resins.		1. Place 3 increments of resin of 2mm each.
			2. Light cure 40 s per increment at 1200 mW/cm^2^.
	Fibres: Silica (20 nm), zirconia fibres (4-11 nm) and cluster zirconia/silica). Inorganic fibres 72.5-87.5wt%.

### Sample characteristics and preparation

Healthy human third molars were extracted 3 months^15^ before the experimental study. Remnants of soft tissue or bacterial plaque were removed with a dental ultrasonic device (DTE D5 LED, Woodpecker). They were then stored in 70% ethanol at 4°C for 2 weeks.^6^ Finally, they were preserved in deionised distilled water until use. The water in which the teeth were stored was changed every 3 days.

**Fig. 2:**
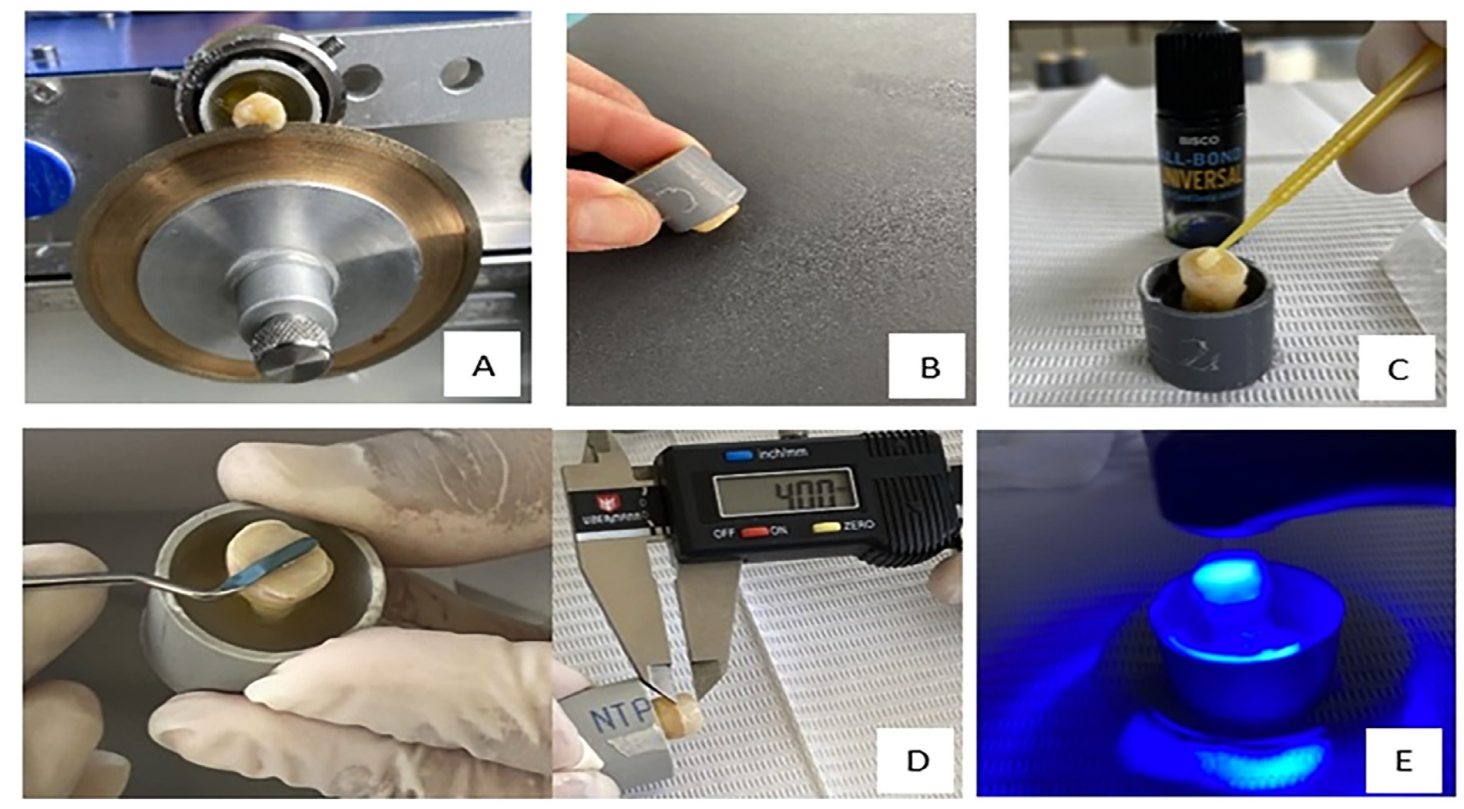
A, Cutting of molars in occlusal third. B, Dentin surface polishing. C, Placement of adhesive system. D, Placement of resin in 2 mm incremental layers. E, Light curing of composite resin.

The teeth were individually placed in 25 × 15 mm PVC (polyvinyl chlorate) tubes, fixed with adhesive wax (Koriwax) and filled with self-curing acrylic (Reliance, Dental Mfg. Co.), leaving the tooth fixed and parallel to the tube. The occlusal third of the crown was removed using a high-precision cutting machine OCP 100 (ODEME) and a diamond disk (#7020, KG Sorensen) at a speed of 300 rpm with constant cooling (Figure 2A). Flat surfaces were prepared on medium coronal dentin with a residual thickness of approximately 2.5 mm. The dentin surface was polished with 600 grit water sandpaper for 60 seconds to obtain a standardised dentin smear (Figure 2B). The tubes were labelled and randomly assigned to the 4 groups for each universal adhesive. Immediately, the adhesive system was actively applied with a microbrush (Microbrush® Plus and Tube Series) according to the manufacturer's instructions (Figure 2C and Table 1). Two increments (2 mm) of Filtek Z350 resin colour A1 (3M ESPE) were then placed (Figure 2D) and each increment was cured for 20 seconds with a light curing device (LED) (Valo®, Ultradent©) at an intensity of 1000 mW/cm^2^(Figure 2E).^14^^,^^16^ All specimens were subjected to 10,000-cycle thermocycling ageing process with an immersion time of 30 seconds and an inter-bin transfer time of 3 seconds between 5 and 55°C prior to microtensile testing (µTBS).^5^^,^^17^

### Microtensile bond strength test

**Fig. 3:**
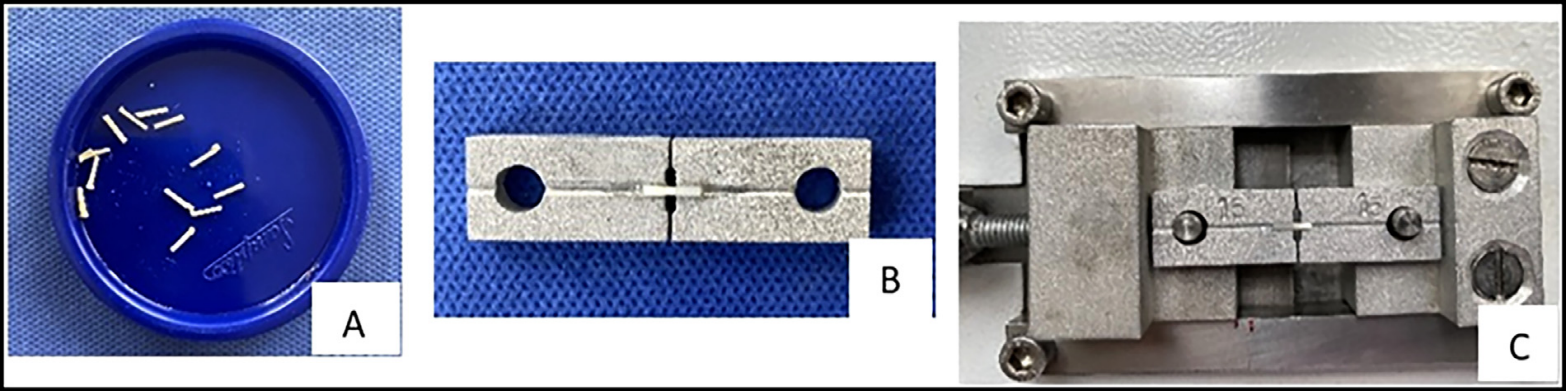
A, Obtaining composite dentin-resin beams. B, Beams fixed to metal bases. C, Machine for microtensile testing.

Each restored tooth was sectioned in the vestibulo-palatal direction across the restoration interface of the tooth using the OCP 100 high-precision digital cutting machine (ODEME) to obtain sections approximately 0.98 mm thick. It was then sectioned in the mesial-distal direction to obtain dentin-resin beams with an approximate area of 0.98 mm^2^, as it has been suggested for µTBS testing that the surface area of the evaluated dentin standard area should be in the range of 0.8 to 1 mm^2^. For standardisation purposes, ideally the average area should be as close to 1 mm^2^ as possible; this would make the results clinically understandable.^18^^,^^19^ This area was calculated based on the average of 3 equidistant measurements on each side of the surface. Then, 8 beams per group were randomly selected. A minimum of 32 beams, each approximately 1 mm wide and 8 to 9 mm long, were obtained (Figure 3A). The thickness of each beam was measured with a digital calliper (Uberman digital calliper, Alca company). The obtained specimens were then attached to an aluminium fixture from the resin and dentin ends using a cyanoacrylate-based adhesive (Triz, Soldimix) for microtensile strength testing (µTBS) on the OM 100 universal testing machine (ODEME) (Figure 3B) at a strain rate of 1 mm/min until failure or separation of the resin-dentin beam. Microtensil bond strength values were determined by dividing the applied force (N) by the bond area (N/mm^2^ = MPa) to obtain values in megapascals (MPa) (Figure 3C).

### Failure mode

The failure modes were evaluated under stereomicroscopy (Leica, model S8APO, LAS 3.4 software) at 40× magnification. The specimens were classified as adhesive failure mode (when failure occurs at the adhesive interface), mixed failure mode (failure occurs at the adhesive interface and leaves adhesive or resin residue on the dentin surface), and cohesive failure mode (failure occurs within the dentin or resin, leaving an undercut).^17^

Beams with failure mode classified as cohesive were not included in the study when calculating microtensile bond strength as they do not represent the actual adhesion of the bond interface.^2^

### Statistical analysis

Data for statistical analysis were imported using SPSS (Statistical Package for the Social Sciences Inc.) version 28.0. The mean and standard deviation were used for descriptive analysis. Prior to hypothesis testing, the Shapiro-Wilk test was used to determine whether the bond strength data had a normal distribution, and the Levene test was used to determine homoscedasticity. It was decided to use Welch's robust variance ANOVA test with the Games-Howell post hoc. In addition, Fisher's exact test was used to determine the association between adhesive type and failure mode. The significance level was set at *P* < .05.

## Results

It was observed that the values of microtensile bond strength presented normal distribution, so when comparing the groups with parametric ANOVA tests with Welch's robust test for equality of means, significant differences were obtained in at least 2 groups. The Games-Howell post hoc test for multiple comparisons showed that group D was significantly higher than A (*P* < .001) and B (*P* < .001), but not significantly different from C (*P* = .075). In addition, group B and C were significantly larger than group A (*P* < .001 and *P* < .001, respectively), but there was no significant difference between group B and C (*P* = .132) (Table 2).

**Table 2:** Descriptive values and comparison of microtensil bond strength (MPa) according to the groups evaluated.

Groups	n	Min	Max	Mean	SD	SE	95% CI		*P	†*P*
							LI	LS		
**A**	8	7.43	12.41	10.21 ^X^	1.49	0.53	8.97	11.45	.806	<.001
**B**	8	13.08	14.94	13.85 ^Y^	0.60	0.21	13.35	14.35	.604	
**C**	8	13.24	17.26	15.34 ^Y,Z^	1.60	0.57	14.00	16.68	.320	
**D**	8	15.16	24.02	18.84 ^Z^	3.17	1.12	16.19	21.49	.055	

95% CI, 95% confidence interval; LL, lower limit; Max, maximum; Min, minimum; n, sample; SD, standard deviation; SE, standard error of the mean; UL, upper limit.

⁎ Based on Shapiro-Wilk Normality test (*P* > .05, normal distribution).

† Based on ANOVA of Welch's robust test of equality of means. X, Y, and Z: Different letters in the same column as the mean indicated significant differences based on Games-Howell post hoc test. A: Palfique Universal Bond; B: Single Bond Universal; C: All-Bond Universal y D: One Coat 7 Universal.

**Fig. 4:**
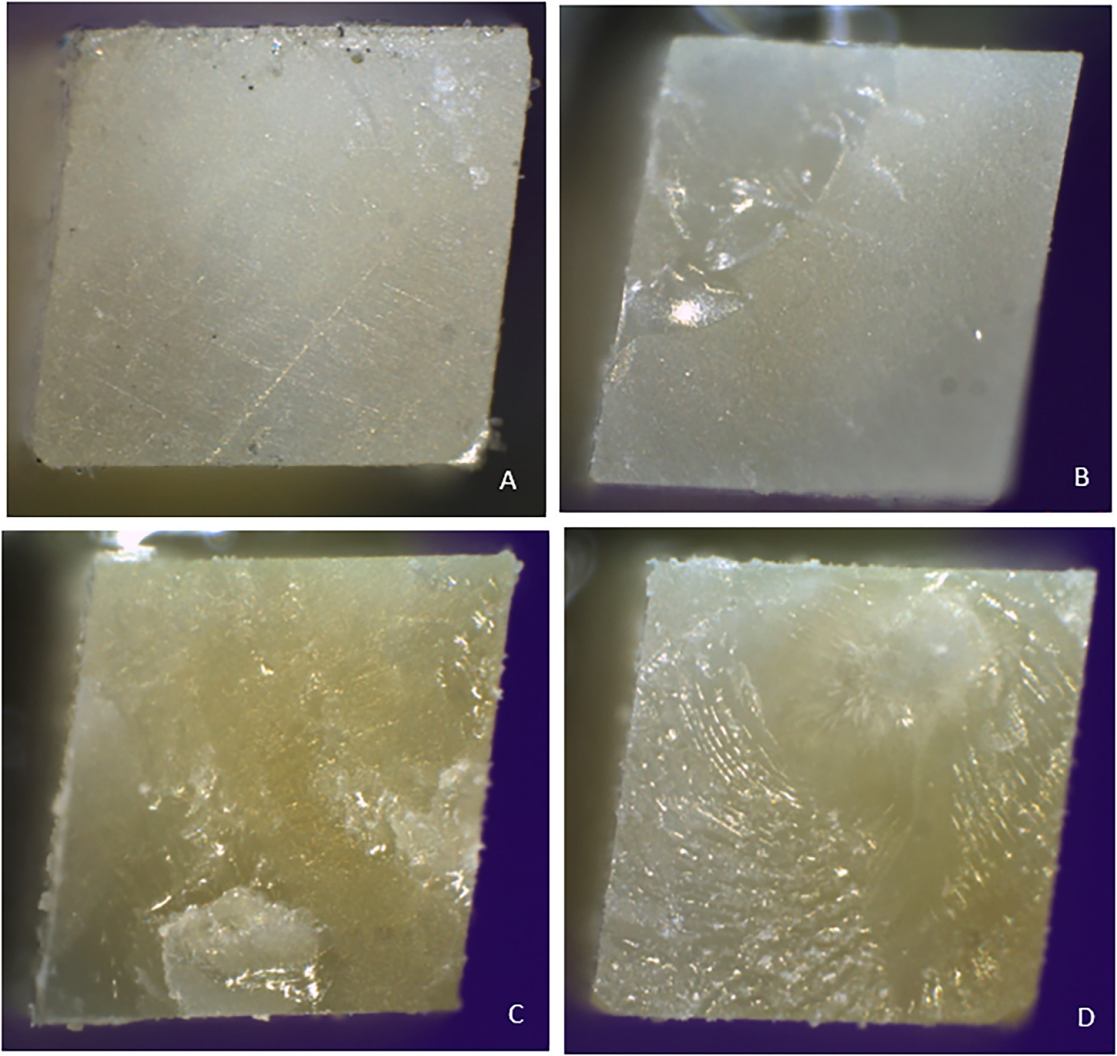
Failure modes assessed with stereomicroscopy. A, Adhesive failure in the group treated with Palfique Bond. B-D, Mixed failure in groups treated with Single Bond, All Bond and One Coat 7.

When analysing the dentin-resin interface, the adhesives were significantly associated with an adhesive or mixed failure mode (*P* = .008). Palfique Bond was significantly associated with an adhesive failure mode (*P* < .05), while Single Bond, All-Bond and One Coat 7 were significantly associated with a mixed failure mode (*P* < .05) (Table 3 and Figure 4).

**Table 3:** Association between failure mode and adhesive used.

Failure mode	Adhesives				P*
	Palfique Bond (A)	Single Bond (B)	All-Bond (C)	One Coat 7 (D)	
	f (%)	f (%)	f (%)	f (%)	
**Adhesive**	6_a_ (66.7)	2_b_ (22.2)	0_b_ (0.0)	1_b_ (11.1)	.008*
**Mixed**	2_a_ (8.7)	6_b_ (26.1)	8_b_ (34.8)	7_b_ (30.4)	
***n***	8 (25.0)	8 (25.0)	8 (25.0)	8 (25.0)	

f: absolute frequency; n: sample size per group.

⁎ *P* < .05 (significant association, based on Fisher's exact test); a, b: Different letters in each row indicate significant differences (*P* < .05) according to Z-test.

## Discussion

Different universal adhesive systems containing functional monomers such as 10-MDP and 3D-SR interact with dentin in different ways, directly affecting the adhesive bond.^1^ In the present study, universal adhesive systems containing 10-MDP monomer such as Single Bond, All Bond and One Coat 7 had better bond strength to dentin after artificial aging than Palfique Universal Bond containing 3D-SR monomer. This is explained by the interaction between the unsaturated 10-MDP monomer and the acid monomer associated with HEMA, which improves the wetting of the tooth surface and the chelation of calcium ions to dentin.^2^^,^^20^^,^^21^ Therefore, based on the results obtained, the null hypothesis was rejected.

Dentin is known to be a less favourable substrate than enamel for bonding with composite resins due to its high organic content, the presence of periprocess fluid and Tomes fibrils in the dentinal tubules.^22^^,^^23^ Bond stability at the dentin-resin interface plays an important role in the clinical performance of composite restorations. Bonding strategies include total etch (each and rise), self-etch, or selective etch when universal adhesive systems are used. Regardless of the strategy used, the goal is to form a hybrid layer as a result of adhesive impregnation and to achieve a durable bond between the composite resin and dentin, which is still a challenge.^24^^,^^25^

In the present study, the samples were subjected to 10,000-cycle thermocycles ageing process between 5 and 55°C, which, according to several authors such as Gale et al,^26^ Cengiz et al,^5^ Cayo et al,^27^ Cahuana et al,^28^ and Alcantara et al,^29^ represent acceptable standardised conditions for laboratory studies that would simulate 1 year of clinical aging. Furthermore, it was decided not to compare it with time 0 (without thermocycling), because different studies indicate that thermocycling allows easy, fast and realistic prediction of the longevity of dental materials bonded to the tooth to assess their mechanical and structural deterioration characteristics during artificial clinical ageing, simulating to some extent the complex environment of the oral cavity.^5^^,^^14^^,^^27^, ^28^, ^29^, ^30^ In terms of in vitro simulations and laboratory evaluations, thermal cycling in dynamic tests is one of the most widely used and accepted procedures, as it better mimics the oral conditions to which dental composites are clinically subjected, which would be valuable in predicting the long-term performance and clinical success of biomaterials.^30^^,^^31^

It should be noted that the storage time in water would cause a change in the structure of the composite resin, reducing the frictional forces between the polymer chains. This process would explain the reduction in bond strength to dentin compared to the immediate bond strength. Another factor to consider is the effect of dentin moisture, which could contribute to the dilution of the adhesives.^5^^,^^28^ On the other hand, it has been reported that thermal cycling may affect bond strength results, as some studies reported that bond strength without thermal cycling showed higher values for adhesives such as Single Bond Universal, All Bond Universal, Clearfil Universal Bond, Palfique Bond and lower values for the same adhesives under 10,000 thermal cycles.^1^^,^^17^^,^^31^^,^^32^

One of the major problems in adhesive dentistry is to find a versatile, long-lasting adhesive system that provides adequate bond strength to both enamel and dentin and does not require a delay in the technique.^5^^,^^33^ For this reason, so-called “universal” or “multimodal” adhesive systems have been developed that allow the dentist to choose between a total-etch, self-etch or selective approach.^34^ In the present study, universal adhesives containing monomers such as 10-MDP and 3D-SR, which form a chemical bond to the apatite, were used to protect the hybrid layer from hydrolytic degradation.^33^

Based on the results of the present study, the One Coat 7 Universal, All Bond Universal and Single Bond Universal adhesive systems containing the 10-MDP functional monomer achieved the highest μTBS values. These results were in agreement with those of Silva et al^1^ and Wang et al^8^ when they evaluated the bond strength of universal adhesive systems containing 10-MDP versus others such as 3D-SR and glycerophosphate dimethacrylate, arguing that the stable chemical bond created by 10-MDP to hydroxyapatite calcium is advantageous for the durability of the bond between the adhesive and dentin, with and without thermocycling, because the long hydrophobic alkyl chain of 10-MDP does not come into contact with water, and the stable nano-layers formed by the bonding of 10-MDP to calcium provide an adhesive interface against biological degradation. It is worth mentioning that in the present study higher values were obtained with the Single Bond Universal adhesive system compared to the Palfique Universal adhesive, since the former contains, in addition to 10-MDP, a copolymer of polyalkenoic acid (VitrebondTM), the main component of glass ionomers, whose 50% of carboxyl groups chemically and spontaneously bind to hydroxyapatite, being able to form ionic bonds with calcium.^17^^,^^35^ The fundamental difference between polyalkenoic acid and 10-MDP is that the latter is a single monomer that upon polymerisation becomes a hydroxyapatite-bonded polymer, whereas polyalkenoic acid is a polymer with a variety of carboxyl functional groups that, attached to the polymer backbone, can “grab” calcium at different sites.^7^

Nikaido et al,^3^ in their study, argued that the soluble stability of the 3D-SR functional monomer is probably similar to that of 10-MDP, and argued that 3D-SR could partially self-organise within the adhesive to form monomeric structures with different phosphate groups and polymerising groups capable of interacting with calcium at multiple sites and forming ionic bonds. For Tokuyama Dental, manufacturer of Palfique Universal Bond, as a single-bottle adhesive containing the 3D-SR monomer, it could improve the incomplete polymerisation and lower degree of resin monomer conversion that occurs in some single-bottle self-etching adhesives.^11^ Silva et al^1^ and the present study disagree with Nikaido et al^3^ in that the bond strength to µTBS with Palfique Universal Bond adhesive was not satisfactory. This is probably due to the acid dissociation constant (PKa) of the 3D-SR functional monomer, which is an important parameter in determining the adhesive's potential to demineralise dentin. For reference, the PKa of 10-MDP (Kuraray Medical) is pKa1 = 2.8, pKa2 = 6.9, but that of the 3D-SR monomer is still unknown. Due to the paucity of evidence on the use of Palfique Universal Bond, it is recommended that future research continue to evaluate the bond strength of this adhesive and compare it with other universal systems.

It was found that although both adhesives contain 10-MDP, the bond strength of the One Coat 7 Universal adhesive was significantly higher than that of the Single Bond Universal adhesive. This difference in bond strength is likely due to the quality and concentration of the 10-MDP monomer in terms of purity used in the adhesives. Impurities in the monomer can promote hydrolysis of the monomer, thereby affecting the bond strength in adhesive systems.^36^ This is consistent with the findings of Siqueira et al^37^ who found that One Coat 7 Universal had significantly higher bond strength than Single Bond Universal.

Cengiz et al,^5^ Osorio et al,^38^ and Toledano et al^39^ in their studies reported a higher percentage of adhesive failure mode associated with low bond strength and mixed failure mode with high bond strength, which would suggest a possible relationship between bond strength and failure mode. These results are consistent with the present study, as mixed failure was observed in One Coat 7 adhesive with higher μTBS values, whereas adhesive failure was predominant in Palfique Universal Bond, which had the lowest values. The use of universal adhesive systems such as One Coat 7 Universal, Single Bond Universal or All Bond Universal in direct restorative treatments would simplify the clinical working time by reducing the risk of dentin sensitivity and improving the adhesion between resin and dentin, which is supported by our results as the mixed failure mode was predominant. The favourable bond strength of 10-MDP to dentin would be related to its molecular structure and hydrophobic behaviour at the interface, thus favouring the bonding, success, and longevity of restorations to the dentin substrate.

The present study was able to reduce measurement bias and strengthen its design due to the methodology used for sample size, aging process, sample preparation protocols, products used, among others. However, it should be recognised that, as an in vitro study, it is not possible to extrapolate it to the clinical field, so the results should be taken with caution.^5^ Likewise, it is recommended that this type of research be extended with a scanning electron microscopy study to determine the thickness and characteristics of the hybrid layer formed when self-etching universal adhesive systems are used. It is also suggested to extend the sample, taking as reference the statistics obtained in the present study, in order to generalise the results under statistical inference.

## Conclusion

The present study concluded that universal adhesive systems and their respective functional monomers interact differently with dentin and directly influence the bond strength. Furthermore, after artificial aging, the One Coat 7 Universal adhesive system stood out with higher bond strength values and a higher chemical interaction with dentin compared to Palfique Universal Bond and Single Bond Universal, but no differences with All-Bond Universal. This allows suggesting One Coat 7 Universal and All-Bond Universal as good alternatives for future randomised clinical trials.
